# Prediction of Coronary Artery Disease Using Machine Learning Techniques with Iris Analysis

**DOI:** 10.3390/diagnostics13061081

**Published:** 2023-03-13

**Authors:** Ferdi Özbilgin, Çetin Kurnaz, Ertan Aydın

**Affiliations:** 1 Department of Electrical and Electronic Engineering, Giresun University, Giresun 28200, Turkey; 2 Department of Electrical and Electronic Engineering, Ondokuz Mayıs University, Samsun 55139, Turkey; 3 Faculty of Medicine, Department of Cardiology, Giresun University, Giresun 28200, Turkey

**Keywords:** iris, iridology, coronary artery disease, diagnosis, machine learning

## Abstract

Coronary Artery Disease (CAD) occurs when the coronary vessels become hardened and narrowed, limiting blood flow to the heart muscles. It is the most common type of heart disease and has the highest mortality rate. Early diagnosis of CAD can prevent the disease from progressing and can make treatment easier. Optimal treatment, in addition to the early detection of CAD, can improve the prognosis for these patients. This study proposes a new method for non-invasive diagnosis of CAD using iris images. In this study, iridology, a method of analyzing the iris to diagnose health conditions, was combined with image processing techniques to detect the disease in a total of 198 volunteers, 94 with CAD and 104 without. The iris was transformed into a rectangular format using the integral differential operator and the rubber sheet methods, and the heart region was cropped according to the iris map. Features were extracted using wavelet transform, first-order statistical analysis, a Gray-Level Co-Occurrence Matrix (GLCM), and a Gray Level Run Length Matrix (GLRLM). The model’s performance was evaluated based on accuracy, sensitivity, specificity, precision, score, mean, and Area Under the Curve (AUC) metrics. The proposed model has a 93% accuracy rate for predicting CAD using the Support Vector Machine (SVM) classifier. With the proposed method, coronary artery disease can be preliminarily diagnosed by iris analysis without needing electrocardiography, echocardiography, and effort tests. Additionally, the proposed method can be easily used to support telediagnosis applications for coronary artery disease in integrated telemedicine systems.

## 1. Introduction

Approximately 17.9 million people die annually due to cardiovascular disease, about 30% of global deaths [1]. The American Heart Association reports that about half of American adults are affected by heart disease. If precautions are not taken, then by 2030, the global death toll is projected to rise to 22 million [2]. Coronary Artery Disease (CAD) has the highest mortality rate among cardiovascular diseases [3]. Coronary arteries are the arteries on the surface of the heart that supply the heart with blood. The blood pumped by the heart first carries oxygen to the heart muscles through the coronary arteries. Three main coronary arteries exist: the left anterior descending artery, the left circumflex artery, and the right coronary artery. CAD occurs due to the decrease or complete cessation of blood flow to the heart muscle caused by the hardening of these coronary arteries [4,5]. The main cause of hardening (plaque formation) in the vessels is the accumulation of fatty or fibrous materials on the inner walls of the vessels, also called atherosclerosis. Plaques are mostly composed of lipids, cholesterol, and apoptosis residues which reduce blood flow, increasing the risk of blood clot formation and embolization [6].

This study defines patients with CAD as patients who are 18 years or older and have at least one clinical scenario of a chronic coronary syndrome (CCS) based on the 2019 European Society of Cardiology (ESC) guidelines for the diagnosis and treatment of CCS. The clinical scenarios for CAD include: (i) patients with suspected CAD and stable anginal symptoms and/or dyspnea, (ii) patients with newly onset heart failure (HF) or left ventricular (LV) dysfunction and suspected CAD, (iii) asymptomatic and symptomatic patients, or recently revascularized patients with stable symptoms less than one year after ACS, (iv) asymptomatic and symptomatic patients more than one year after the diagnosis of CCS or revascularization, (v) patients with angina and suspected vasospastic or microvascular disease, and (vi) asymptomatic participants detected to have CAD during routine screenings.

A series of laboratory tests and imaging methods are used to diagnose CAD. The diagnosis is made by evaluating the patient’s complaints, family history, risk factors, and the results of physical examination findings. To diagnose CAD, blood tests, electrocardiography (ECG), effort tests, Holter tests, and echocardiography (ECHO) are commonly used tests [7,8]. The onset of symptoms in patients with CAD can range from simple nonspecific chest pain to a direct heart attack or even death. Neglected findings can lead to a heart attack; even if the patient does not die, severe damage to the heart muscle can occur. Therefore, early diagnosis is very important in CAD [4]. In recent years, the iris, which contains many nerve endings, has been used for the early diagnosis of diseases. The iris contains approximately 28,000 neural networks communicating between the brain and organs [9]. If an organ is not functioning properly, information is sent to the brain about this situation, which is reflected in the iris as a change in pattern, color, or characteristic feature. Iridology is the study of the changes in pattern, texture, color, and structure that occur in the special regions of the iris and their relationship with various diseases. As a result of various studies within the field of iridology, iris maps were created that show the regions in the iris that are related to specific organs and tissues. Bernard Jensen finalized the Iris map, which consists of 166 sections, 80 on the right and 86 on the left [10,11].

### 1.1. Related Work on IRIS

When reviewing the literature, iridology studies investigate the anatomical changes in specific areas of the iris, which are typically caused by functional changes in a particular organ or tissue [12]. Ma et al. discovered with significant accuracy that diseases can be diagnosed using geometric features such as the size of the pupil, shape, and shape of the iris [13]. Samant and Agarwal conducted a study to diagnose diabetes using various machine-learning techniques by analyzing the texture of the iris pancreatic region. The study found an accuracy rate of around 90% [14]. Similarly, many other models for diagnosing diabetes have been proposed by researchers in recent years [15,16,17]. Rehman et al. proposed an iridology-based approach for diagnosing chronic liver disease [18]. They found that iris analysis combined with the ensemble learning method had an accuracy rate of approximately 98%. In the literature, there are studies on diseases of organs such as the kidney [19] and brain [20] using iridology, and there are various studies on cholesterol values in the blood [12,21,22,23]. In line with these studies, iridology has been shown to be effective in the non-invasive early diagnosis of diseases. However, there is a limited amount of research on the use of iris analysis for the early diagnosis of heart diseases. Various researchers around the world have made significant discoveries in non-invasive image processing and artificial intelligence-based diagnosis by using iris images related to the heart, which is a vital organ for maintaining life functions. Gunawan et al. [24] proposed a method for detecting coronary artery disease using the Support Vector Machines (SVM) classifier with five Gray-Level Co-Occurrence Matrix (GLCM) features. In their study involving 250 volunteers, the features of 100 volunteers were used as test data, and the Gaussian kernel SVM classifier achieved 91% accuracy in detecting coronary artery disease. Putra et al. [25] developed a system with 90 volunteers utilizing iris analysis to detect cardiac issues. They employed the Principal Component Analysis (PCA) and Gray-Level Co-Occurrence Matrix (GLCM) methods to extract features in the system they developed, and they performed the classification process using neural networks. They achieved a classification accuracy of 77.5% for the test data using GLCM features, and they achieved 90% accuracy using PCA features. The PCA feature extraction method and SVM classifier were utilized in the method proposed by Permatasari et al. [26]. The highest accuracy achieved was reported to be 80%. Kusuma et al. [27] proposed a model for detecting cardiac abnormalities by acquiring and using iris images with a mobile-based system. The ratio of black and white pixels obtained after converting the analysis region to black and white format was used as a feature. The accuracy performance value for the test data, as classified by the thresholding method, was measured at 83.3%. These studies demonstrate the effectiveness of using iridology for the diagnosis of CAD.

### 1.2. Research Gaps of Previous Work on IRIS/CAD

When studies in the literature are examined, it is seen that various methods are used to diagnose heart diseases via the iris early. However, it appears that no specific heart disease has been evaluated in depth. These studies follow a standard procedure, including finding the iris positions, performing the rectangular transformation, determining the analysis region, extracting the features from the analysis region, and classification. The differences in the studies begin after the determination of the analysis region. When the studies are examined at this stage, it is seen that the sub-components were formed by applying the wavelet transform to the analysis region, and although successful results were obtained in the studies conducted for the diagnosis of diabetes, this method has not been tested for heart diseases. In this study, more comprehensive and qualified results were obtained compared to the existing studies for the diagnostics of CAD by increasing the number of features to be extracted using the wavelet transform and the number of classifiers.

### 1.3. Contribution of This Paper

In this study, a new diagnostic approach is proposed using iris images for the non-invasive detection of CAD. The data used in the study were collected from 198 volunteers, including 94 individuals with CAD and 104 control individuals, from the Cardiology Polyclinic of Giresun University Health Practice and Research Hospital. The study includes a feature selection method based on wavelet transform, resulting in 136 features, including statistical, GLCM, and Gray-Level Run Length Matrix (GLRLM) features. According to their rank values, the best 25, 50, and 75 features were selected using the Relieff method. A total of 22 classifiers belonging to the Decision Trees (DT), Naive Bayes (NB), Support Vector Machines (SVM), k-Nearest Neighbor (kNN), and Neural Networks (NN) families, which are commonly used for classification, were applied. The performance metrics calculated in the study indicate that the proposed model is more successful in detecting CAD than existing models. Detailed comparisons and evaluations are provided in the Results and Discussion section.

The main contributions of this study can be summarized as follows:

- A novel diagnostic approach is proposed for the non-invasive detection of CAD using iris images.
- The Relieff feature selection method based on wavelet transform is introduced, resulting in 136 features including statistical, GLCM, and GLRLM features.
- A comparison is made between different classifiers, such as DT, NB, SVM, kNN, and NN, and the best-performing classifier is identified.
- The proposed model was compared with existing models and was more successful in detecting CAD.

## 2. Materials and Methods

**Figure 1:**
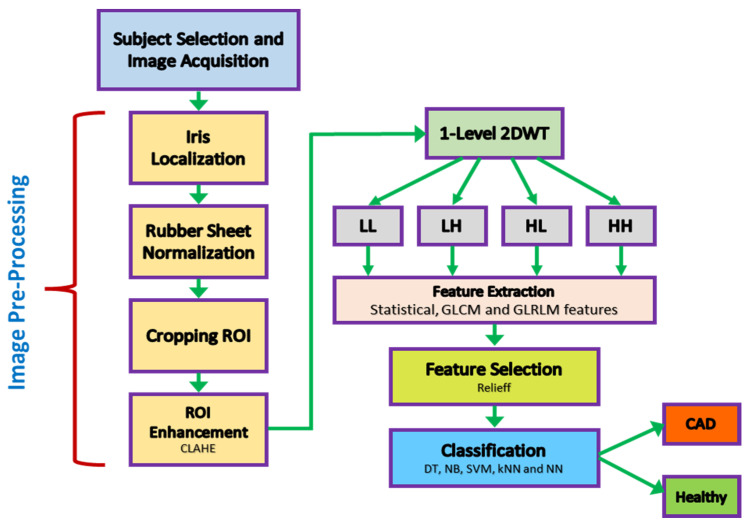
Proposed methodology.

In the study methodology, a standard design was carried out to diagnose CAD through a non-invasive procedure. The flow chart of the study is shown in Figure 1.

### 2.1. Subject Selection for Data Acquisition

**Figure 2:**
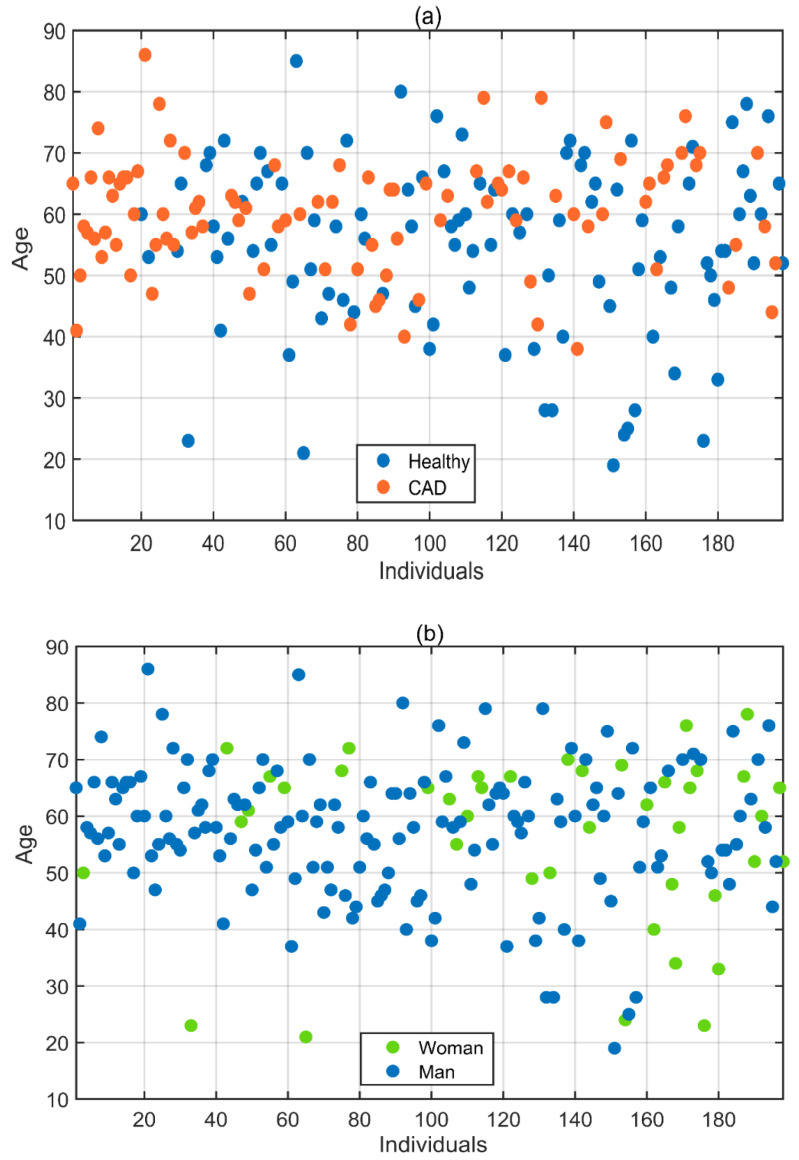
(**a**) Health status and (**b**) gender information displayed according to the ages of the volunteers.

In this study, the dataset was created by collecting iris images from 198 subjects with the volunteers’ consent and with the assistance of relevant doctors from the Giresun University Health Practice and Research Hospital Cardiology Polyclinic. Ethics committee approval was obtained for data collection per the decision of Samsun University Clinical Research Ethics Committee, numbered SUKAEK-2022 12/21, dated 23 November 2022. Out of the 198 volunteers, 94 were diagnosed with CAD, while 104 were healthy individuals without the disease. The incidence of CAD varies according to gender, with it being more common in men [1]. As a result, the proportion of men among the volunteers included in the study is higher than that of women. Of the volunteers aged between 19 and 86 who participated in the study, 156 were men and 42 were women. Table 1 and Figure 2 provide detailed information about the age, gender, and health status of the volunteers.

### 2.2. Eye Image Acquisition

**Figure 3:**
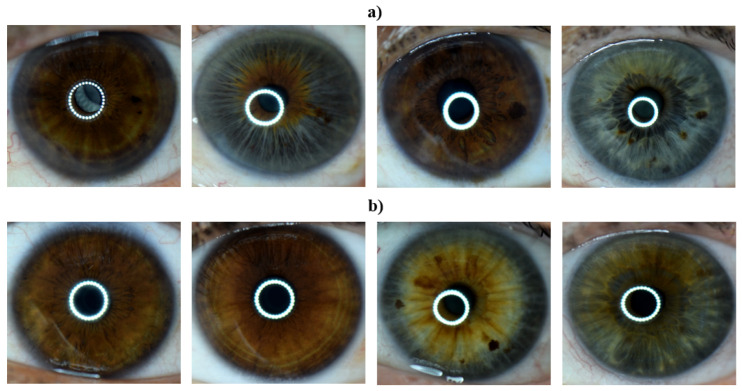
Iris images of the (**a**) CAD and (**b**) control group in the data set.

Left eye images of the subjects labeled as having CAD and of those labeled as healthy by their respective doctors were collected. Eye images were taken using a Nikon D3300 DSLR camera with a Nikon AF-S DX Micro Nikkor 85 mm F/3.5G VR lens and with macro ring flash illumination. The resulting images were 6000 × 4000 in size and had a resolution of 24 megapixels. Example images for both healthy and CAD volunteers are provided in Figure 3.

### 2.3. Eye Image Pre-Processing

**Figure 4:**
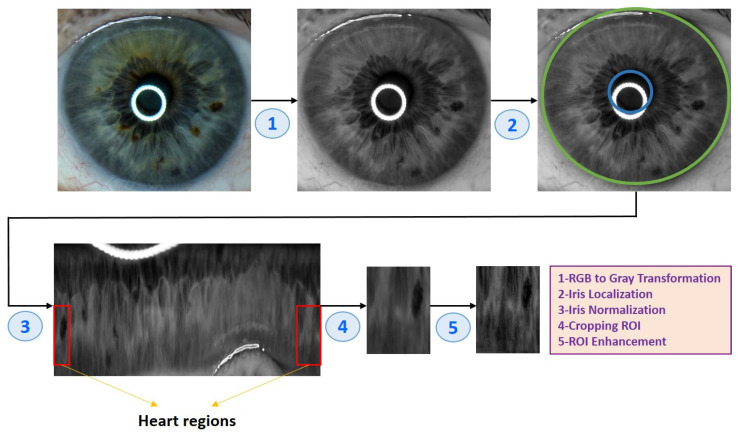
An example of the pre-processing process used in the study.

After obtaining the eye images, they needed to go through several pre-processing steps to prepare them for analysis. Algorithm 1 and Figure 4 illustrate the eye image pre-processing process step-by-step.
**Algorithm 1** Eye image pre-processing algorithm**(1) Input: Eye image** **(2) Iris localization from the eye image**  (a) Localization pupil using Daugman’s Integral Differential Operator  (b) Localization iris using Daugman’s Integral Differential Operator **(3) Iris normalization using Daugman’s rubber sheet Technique**  - Normalized iris becomes a fixed size: 360 × 720 **(4) ROI cropped according to the iris map in Figure 4**  - The ROI size is 190 × 120 **(5) ROI enhancement using the CLAHE method** **(6) Output: ROI image**

The techniques used for the image pre-processing process are as follows:

#### 2.3.1. Iris Localization

At this stage, the pupil and iris positions were determined from the image. The iris positions in the image converted to the gray format were determined using the integral differential operator (IDO) [28]. The IDO method can accurately determine the inner and outer borders of the iris by using different values of pupil and sclera color. The mathematical expression of the method is provided in the equation below.

(1) $$\underset{r,x_{0},y_{0}}{\max}\left|G_{\sigma }\left(r\right)\frac{\begin{array}{c} \partial \end{array}}{\partial r}\oint_{r,x_{0},y_{0}}^{}\frac{I(x,y)}{2\pi r}ds\right|$$

Here, the expression I(x, y) denotes the color value of the (x, y) position in the image I. x_0_ and y_0_ represent the coordinates of the potential center point, and the symbol r represents the distance to the potential center point. G_σ_ represents the Gaussian function with σ standard deviation.

#### 2.3.2. Iris Normalization

**Figure 5:**
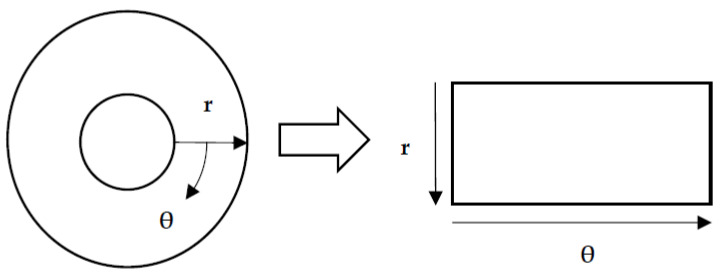
Iris normalization.

The normalization process was the next step after determining the iris’s inner and outer positions. The iris was transformed into a rectangular format in the normalization process, standardizing it and making it easier to analyze. As a result of the normalization process, the rectangular iris image was resized to a fixed size of 360 × 720. Daugman’s rubber sheet method, as shown in Figure 5, is one of the most commonly used normalization methods, and it was used in this study.

The remapping of the iris image from the *I*(*x*, *y*) cartesian coordinates to the polar representation can be expressed as the following equation.

(2) $$I(x\left(r,\theta \right),y\left(r,\theta \right))\to I(r,\theta )$$

where

(3) $$x\left(r,\theta \right)=\left(1-r\right)x_{p}\left(\theta \right)+rx_{l}(\theta )$$

(4) $$y\left(r,\theta \right)=\left(1-r\right)y_{p}\left(\theta \right)+ry_{l}(\theta )$$

Here, the *I*(*x*, *y*) is the iris region, (*x*, *y*) represents the Cartesian coordinates, (*r*, *θ*) represents the normalized polar coordinates, and *x_p_*, *y_p_* and *x*_l_, *y*_l_ are expressions that denote the pupil and iris boundary coordinates in the *θ* direction.

#### 2.3.3. Region of Interest (ROI)

**Figure 6:**
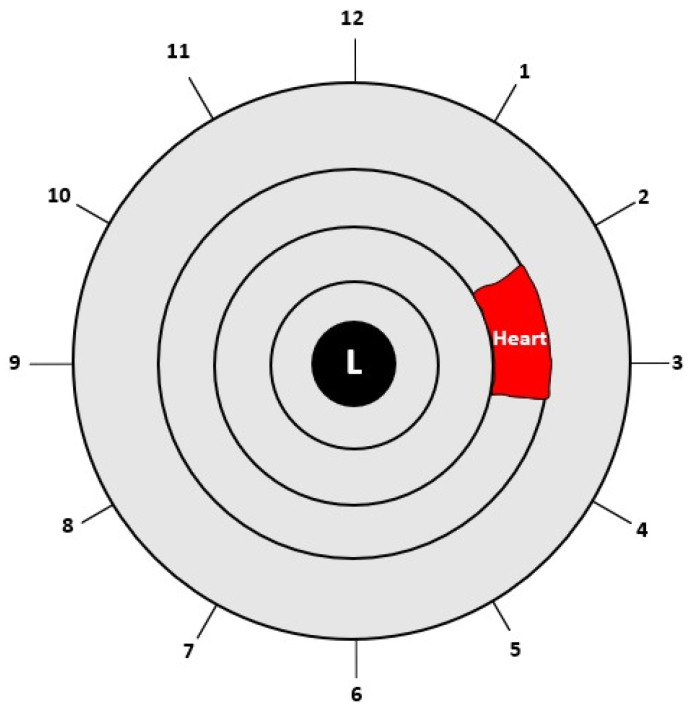
Location of the heart in the left iris [10].

After completing the normalization process, the Region of Interest (ROI) was cropped according to the heart region in the left iris in the iris map shown in Figure 6. The heart region is located in the left iris between the 2 and 4 o’clock positions. After converting the circular iris image to a fixed-size rectangle, the heart region in the iris was cropped.

#### 2.3.4. Enhancement of ROI

Histogram equalization is a commonly used image enhancement technique due to its high performance and simplicity. It redistributes the probabilities of the occurrence of gray-levels so that the histogram of the output image is closer to a uniform distribution. Although the method generally gives good results, it may not achieve the desired improvement in images with darker or lighter colored pixels than other pixel values. To address this limitation, instead of using the whole image for equalization, the image was divided into certain regions, and the histogram equalization of the regions increased image improvement performance. The Contrast Limited Adaptive Histogram Equalization (CLAHE) method [29] was used for this purpose. In this study, the CLAHE method was used for ROI correction.

### 2.4. Iris Feature Extraction

Because the iris contains many blood vessels and nerves, it has a very rich structural pattern. Many researchers have extracted features from the iris using various methods such as the Gabor Filter, Hilbert Transform, and Discrete Wavelet Transform (DWT). In this study, DWT transformation was used for feature extraction. The process of feature extraction is outlined in Algorithm 2.
**Algorithm 2** Feature extraction process**(1) Input: ROI Image** **(2) Perform 1 Level 2D-DWT to ROI image**  - Four sub-bands occur (cA, cV, cD, cH) **(3) Extract features from sub-bands**  (a) Extract 5 first-order statistical features as shown in Table 2  (b) Extract 22 GLCM-based features as shown in Table 3  - Formation of the 8 × 8 GLC matrix using θ = (0^0^, 45^0^, 90^0^, 135^0^) with d = 1. Values for each direction are found and averaged  (c) Extract 7 GLRLM-based features as shown in Table 4  - Formation of the GLRL matrix using θ = (0^0^, 45^0^, 90^0^, 135^0^) with quantize level = 16. Values for each direction are found and averaged **(4) Fusion of features (5 statistical + 22 GLCM + 7 GLRLM = 34 features for each sub-band)** **(5) Output: feature vector with 136 features**


**Figure 7:**
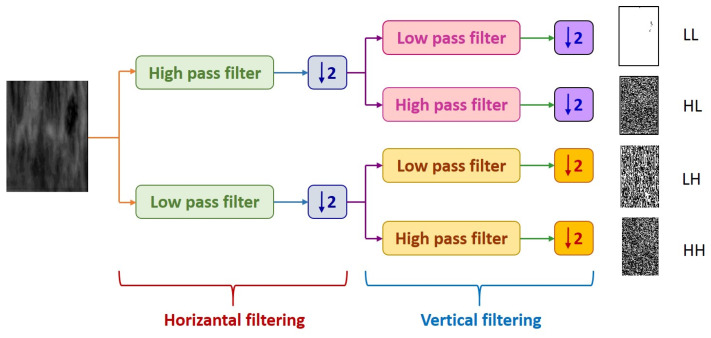
1-Level DWT decompositions.

DWT decomposes an image into four subsampled images, as shown in Figure 7, namely the approximation (LL), horizontal (HL), vertical (LH), and diagonal (HH) images. The input image of size N × N is divided into four sub-images, each of size N/2 × N/2. Each sub-image contains information from different frequency components [30].

In Figure 7, the LL sub-band was obtained by applying low-pass filtering to both rows and columns, resulting in an image with less noise than the other sub-bands. The HH band was obtained by applying high-pass filtering in both directions, and it contains higher frequency components than the other bands. The HL and LH sub-bands were obtained by using low-pass filtering in one direction and high-pass filtering in the other. The LH sub-band mostly contains vertical detail information corresponding to horizontal edges, while the HL sub-band contains horizontal detail information corresponding to vertical edges. The HL, LH, and HH sub-bands add high-frequency detail to the approximate image. The image is typically decomposed multiple times using the DWT, usually starting with the LL band [31]. 

**Figure 8:**
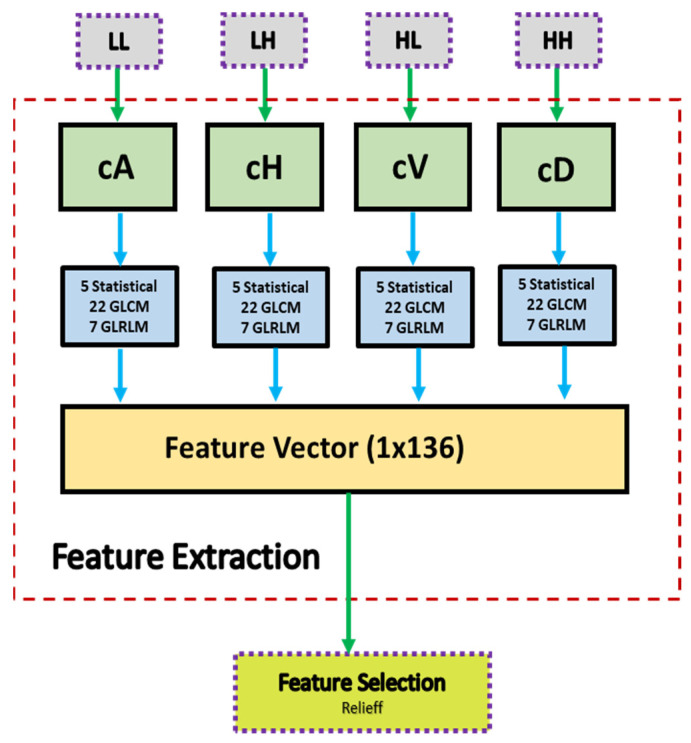
Block diagram of the feature extraction process.

A block diagram of the feature extraction process is shown in Figure 8. In Figure 8, cA describes the approximation coefficients matrix, and cH, cV, and cD describe the detail coefficients’ matrices (horizontal, vertical, and diagonal, respectively). A total of 34 features were extracted for each of the four coefficients’ matrices (cA, cH, cV, cD). These features included five statistical features, 22 GLCM (Gray Level Co-occurrence Matrix) features, and 7 GLRLM (Gray Level Run Length Matrix) features. At the end of the feature extraction process, 136 feature vectors (34 for each region) were obtained.

**Figure 9:**
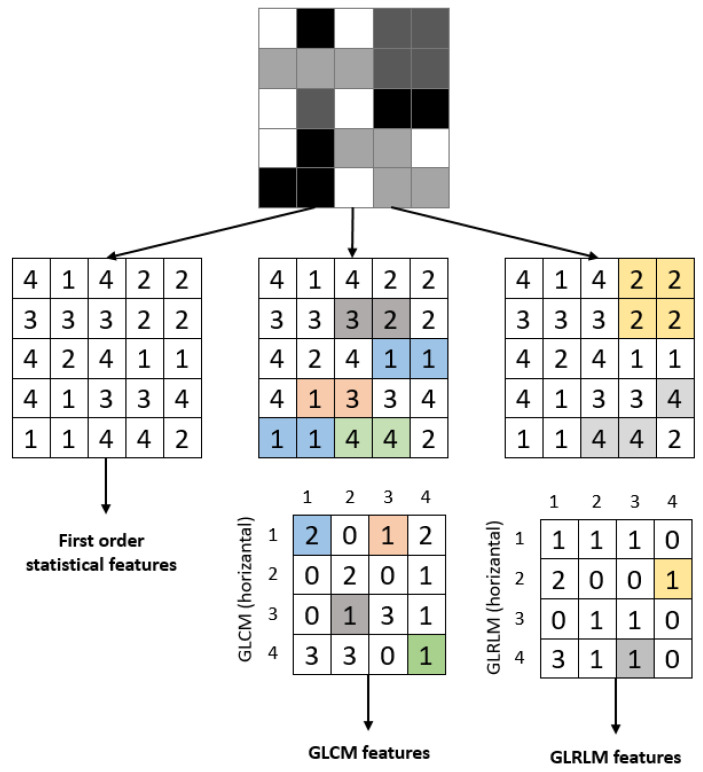
Calculating first-order statistical and textural features for a sample 5 × 5 image; whereas GLCM relies on pixel pairs (distance = 1, θ = 0°), GLRLM relies on runs.

This study used a 1-level DWT decomposition to analyze the ROI image. Statistical features and features obtained using GLCM and GLRLM were extracted for each sub-band. Figure 9 provides an example of extracting features for a sample image. The attributes of the extracted features are described in the following headings.

#### 2.4.1. Statistical Features

The study calculated and used the ROI’s five first-order statistical features: mean density, standard deviation, entropy, skewness, and kurtosis. The mathematical expressions for these parameters obtained from the gray-level ROI are provided in Table 2. Five statistical features were obtained for each sub-band.

**Table 2:** Statistical features.

Feature Name	Formula	Feature Name	Formula
Mean intensity	$\frac{1}{N}\sum_{i=1}^{N}X(i)$	Skewness	$\frac{\frac{1}{N}\sum_{i=1}^{N}(X\left(i\right)-\overset{-}{X}{)}^{3}}{{\left(\sqrt{\frac{1}{N}\sum_{1}^{N}(X\left(i\right)-\overset{-}{X}{)}^{2}}\right)}^{3}}$
Standard deviation	${\left(\frac{1}{N-1}\sum_{i=1}^{N}(X\left(i\right)-\overset{-}{X}{)}^{2}\right)}^{1/2}$	Kurtosis	$\frac{\frac{1}{N}\sum_{i=1}^{N}(X\left(i\right)-\overset{-}{X}{)}^{4}}{{\left(\frac{1}{N}\sum_{1}^{N}(X\left(i\right)-\overset{-}{X}{)}^{2}\right)}^{2}}$
Entropy	$\sum_{i=1}^{N_{1}}P\left(i\right).\log_{2}P(i)$		

#### 2.4.2. Gray-Level Co-Occurrence Matrix (GLCM) Features

**Figure 10:**
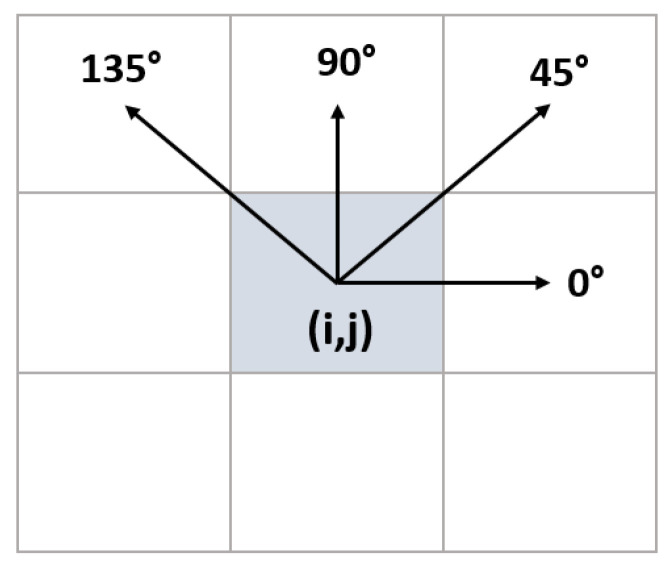
Direction information for neighbor pixel analysis.

Using only first-order statistical approaches is insufficient for detecting and grading textures or patterns in an image. These features provide information about the intensity distribution but do not reveal the relationship between pixels. To gain information about neighboring pixels, GLCM and related features offered by Haralick et al. [32] can be used. GLCM is a gray-level matrix that characterizes, quantifies, and explores the distribution of gray-level intensities. Direction and neighborhood information is used when calculating GLCM. As shown in Figure 10, the 0°, 45°, 90°, and 135° directions were used. When creating the GLCM, the grayscale value of each pixel in the image was calculated as given in Equation (5).

(5) $$P\left(i,j\right)=\frac{P(i,j,d,\theta )}{\sum_{i=1}\sum_{j=1}P(i,j,d,\theta )}$$

After the GLCM of the image was created, the textural features of the image were extracted from this matrix. This study used 22 parameters [32,33,34] to extract features using GLCM. The names, mathematical expressions, and definitions of these parameters are provided in Table 3.

**Table 3:** GLCM features.

Feature Name	Formula	Feature Name	Formula
Auto correlation	$\sum_{i=1}^{N}\sum_{j=1}^{N}\left(i.j\right)p(i,j)$	Information measure of correlation 1	$\frac{HXY-HXY1}{\max(HX,HY)}$
Cluster prominence	$\sum_{i=1}^{N}\sum_{j=1}^{N}{\left(i+j-2u\right)}^{3}p(i,j)$	Information measure of correlation 2	$\sqrt{1-\exp[-2\left(HXY2-HXY\right)]}$
Cluster shade	$\sum_{i=1}^{N}\sum_{j=1}^{N}{\left(i+j-2u\right)}^{4}p(i,j)$	Inverse difference moment	$\sum_{i=1}^{N}\sum_{j=1}^{N}\frac{p(i,j)}{1+\left|i-j\right|}$
Contrast	$\sum_{i=1}^{N}\sum_{j=1}^{N}{\left(i-j\right)}^{2}p(i,j)$	Maximum probability	${max}_{i,j}p(i,j)$
Correlation	$\sum_{i=1}^{N}\sum_{j=1}^{N}\left(\frac{i-{µ}_{x}}{\sigma_{x}}\right)\left(\frac{j-{µ}_{y}}{\sigma_{y}}\right)p(i,j)$	Sum average	$\sum_{k=2}^{2N}kp_{x+y}(k)$
Difference entropy	$-\sum_{k=0}^{N-1}p_{x-y}\left(k\right)log\;p_{x-y}(k)$	Sum entropy	$-\sum_{k=2}^{2N}p_{x+y}\left(k\right)\log p_{x+y}(k)$
Difference variance	$\sum_{k=0}^{N-1}{\left(k-{µ}_{x-y}\right)}^{2}p_{x-y}(k)$	Sum of squares	$\sum_{i=1}^{N}\sum_{j=1}^{N}p{\left(i-µ\right)}^{2}p(i,j)$
Dissimilarity	$\sum_{i=1}^{N}\sum_{j=1}^{N}\left|i-j\right|.p(i,j)$	Sum variance	$\sum_{k=2}^{2N}{\left(k-{µ}_{x+y}\right)}^{2}p_{x+y}(k)$
Energy	$\sum_{i=1}^{N}\sum_{j=1}^{N}p{\left(i,j\right)}^{2}$	Maximal correlation coefficient	$\sqrt{\lambda_{2}(Q\left(i,j\right))}$
Entropy	$-\sum_{i=1}^{N}\sum_{j=1}^{N}p(i,j)log\;p(i,j)$	Inverse difference normalized	$\sum_{i=0}^{N-1}\sum_{j=0}^{N-1}\frac{1}{1+{\left(i-j\right)}^{2}}p(i,j)$
Homogeneity	$\sum_{i=1}^{N}\sum_{j=1}^{N}\frac{p(i,j)}{1+{\left(i-j\right)}^{2}}$	Inverse difference moment normalized	$\sum_{i=0}^{N-1}\sum_{j=0}^{N-1}\frac{p(i,j)}{{\left(1+\frac{\left|i-j\right|}{N}\right)}^{2}}$

The features listed in Table 3 were calculated for the four sub-bands obtained after the wavelet transform. For each wavelet component, the features calculated by considering pixels in four directions and one neighbor distance were averaged. This resulted in the creation of 22 GLCM attributes for each region.

#### 2.4.3. Gray-Level Run Length (GLRL) Matrix Features

The Gray-Level Running Length Matrix (GLRLM) method is based on calculating the number of different gray-level lengths [32]. It is a way of extracting higher-order statistical texture features. A gray-level run is a linear array of adjacent image points with the same gray-level value. The gray-level run length is the number of image points in the array. GLRLM is a two-dimensional matrix and is used for texture feature extraction. In this study, seven attributes, along with their names, mathematical equations, and descriptions, are provided in Table 4, which were used when using GLRLM.

**Table 4:** GLRLM features.

Feature Name	Formula	Feature Name	Formula
Short Run Emphasis (SRE)	$\sum_{i=1}^{G}\sum_{j=1}^{R}\frac{p(\left.i,j\right|\theta )}{j^{2}}/\sum_{i=1}^{G}\sum_{j=1}^{R}\frac{p(\left.i,j\right|\theta )}{1}$	Run Length Non-Uniformity (RLN)	$\sum_{j=1}^{R}{\left(\sum_{i=1}^{G}p(\left.i,j\right|\theta )\right)}^{2}/\sum_{i=1}^{G}\sum_{j=1}^{R}p(\left.i,j\right|\theta )$
Long Run Emphasis (LRE)	$\sum_{i=1}^{G}\sum_{j=1}^{R}j^{2}\times p(\left.i,j\right|\theta )/\sum_{j=1}^{R}p(\left.i,j\right|\theta )$	Low Gray-Level Run Emphasis (LGRE)	$\sum_{i=1}^{G}\sum_{j=1}^{R}\frac{p(\left.i,j\right|\theta )}{i^{2}}/\sum_{i=1}^{G}\sum_{j=1}^{R}p(\left.i,j\right|\theta )$
Gray-Level Non-Uniformity (GLN)	$\sum_{i=1}^{G}{\left(\sum_{j=1}^{R}p(\left.i,j\right|\theta )\right)}^{2}/\sum_{i=1}^{G}\sum_{j=1}^{R}p(\left.i,j\right|\theta )$	High Gray-Level Run Emphasis (HGRE)	$\sum_{i=1}^{G}\sum_{j=1}^{R}i^{2}\times p(\left.i,j\right|\theta )/\sum_{i=1}^{G}\sum_{j=1}^{R}p(\left.i,j\right|\theta )$
Run Percentage (RP)	$\frac{1}{N}\sum_{i=1}^{G}\sum_{j=1}^{R}p(\left.i,j\right|\theta )$		

### 2.5. Feature Selection

Feature selection is an important step in reducing complexity and saving time in machine learning methods for classification problems. It makes classification more reliable by eliminating unnecessary data. Relieff, a widely used filter-based feature selection method, was preferred in this study. The algorithm developed by Kira et al. performs the selection process by weighting the parameters according to their relationship [35]. Kononenko created this algorithm, as the method did not give successful results in datasets with multiple classes [36]. The method selects a sample from the dataset and performs feature selection by creating a model based on the proximity of the sample to other samples in its class and based on its distance from different classes. In this study, the best 25, 50, and 75 features were selected among 136 features obtained from ROI. There were four sub-band images, each containing 34 features. Choosing specific features from each sub-band and including different feature groups can be beneficial in more effectively determining the impact of sub-bands and methods on performance. This approach helps to accurately identify the performance effects of sub-bands and methods.

### 2.6. Classification

In classification, there are two main types: supervised and unsupervised. In supervised classification, the model performance is determined by the test data in models created using labeled data. In this study, 22 classifiers from 5 different classifier families, which are commonly used in literature, were employed.

(a) Decision Trees: Fine, Medium, and Coarse Trees
(b) Naive Bayes: Gaussian and Kernel types
(c) Support Vector Machines with four kernels: Quadratic, Cubic, Fine Gaussian, Medium Gaussian, and Coarse Gaussian
(d) k-Nearest Neighborhood (kNN): Fine, Medium, Coarse, Cosine, Cubic, and Weighted
(e) Neural Networks: Narrow, Medium, Wide, Bilayered, and Trilayered

Although the classifiers mentioned above are commonly used in various fields, the MATLAB Classification Learner application, which includes standard parameters, was used in this study to avoid bias that may occur from manual selection of the parameters. The training and test data were divided into five groups using the fivefold cross-validation technique for the classification process. The performance values were obtained by taking the average of the parameters calculated five times.

### 2.7. Performance Evaluation 

**Figure 11:**
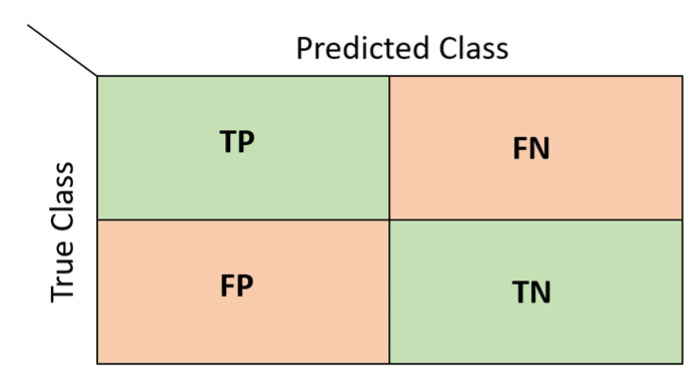
A two-class confusion matrix.

Various evaluation metrics were used to determine the success of the models created during the classification process. These metrics are based on a table called the confusion matrix [37]. Each row of the matrix represents the actual values, and each column represents the predicted values. A two-class confusion matrix and the values it will take are shown in Figure 11.

In Figure 11, TP refers to true positive results, FN refers to false negative results, FP refers to false positive results, and TP refers to true negative results. The metrics used in this study to determine the classification performance using the confusion matrix are listed in Table 5.

Accuracy is the ratio of correct guesses to the total number of values. A high value indicates high accuracy. Specificity is the ratio of correct negative predictions to the total number of negatives. Precision is the ratio of correctly predicted positive observations to the total predicted positive observations, and it measures the accuracy of predictions for positive class. Sensitivity is the ratio of correctly predicted positive observations to all observations in the actual positive class. The F1-score is the harmonic mean of the ratio of true positive values (sensitivity) and precision. It is a measure of how well the classifier is performing. The geometric mean is a metric that measures the balance in classification between majority and minority classes. A low value indicates poor performance in the classification of positive cases, even if it correctly classified negative cases [38,39]. In addition to these metrics, the Receiver Operating Characteristic (ROC) curve was also used to measure performance. The ROC curve is a graphical representation of the performance of a classifier over all possible threshold values. It has False Positive Rate (FPR) on the x-axis and True Positive Rate (TPR) on the y-axis. The Area Under Curve (AUC) is the area under the ROC curve. The AUC value ranges from 0 to 1, and the closer the value is to 1, the better the model’s performance [40].

## 3. Results and Discussion

In this study, iris images of 198 volunteers were analyzed to detect coronary artery disease. The relationship between the 136 features obtained from the iris images and the target variable was first investigated. Then, the performance evaluations obtained from the classification process using the best 25, 50, and 75 features determined by the Relieff feature selection method were presented.

### 3.1. Feature Analysis

**Figure 12:**
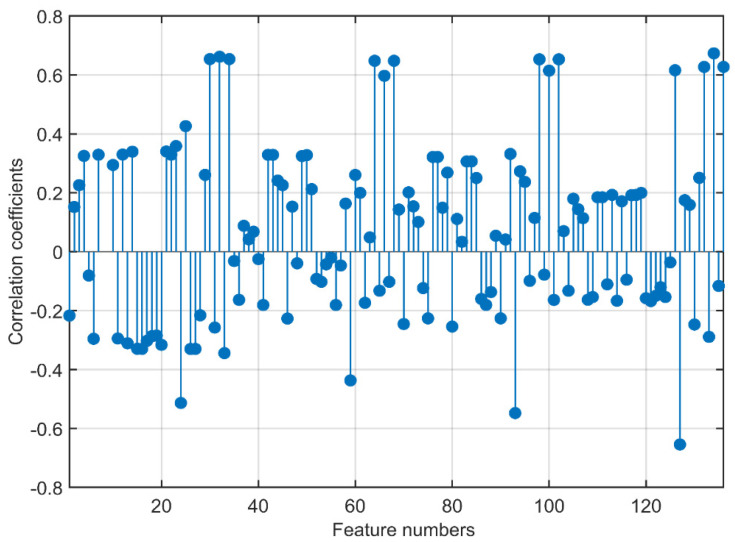
Correlation coefficients show the relationship of the features with the target variable.

The correlation coefficient values showing the relationship of the 136 features obtained from the wavelet transform with the target variable are illustrated in Figure 10. In the ROI, which is divided into four components after the wavelet transform, 34 features, five statistical, 22 GLCM, and seven GLRLM features were extracted for each component. The four components were labeled cA, cH, cV, and cD. In Figure 12, the components and attributes are presented in this order.

The highest correlation value of 0.6734 belonged to the 134th feature, RP, which is a GLRLM attribute of the cD sub-band. There were 13 features in total with a correlation value above 0.6, three features with values between 0.5 and 0.6, two features between 0.4 and 0.5, 24 features between 0.3 and 0.4, and 27 features between 0.2 and 0.3. Among the 10 features with the highest correlation coefficients, there were three features in the cA component, two in the cH component, two in the cV component, and three in the cD component. Nine of these features belonged to GLRLM features, and one of them belonged to a GLCM feature not among the 10 features with the highest 1st-order statistical feature coefficients. Out of the nine GLRLM attributes, LRE 4, LGRE 3, and RP were included twice. RP was the two best attributes. The GLCM attribute also had the highest correlation coefficient. From the high correlation coefficients of the features, it can be seen that the features were evenly distributed among the components obtained from the wavelet transform. It can be observed that the statistical features had lower correlation coefficients compared to the other feature groups, and the highest coefficients were in the GLRLM and GLCM features.

### 3.2. Results after Feature Selection

Before the classification process, the feature selection process was applied. Using the Relieff algorithm, the best 25, 50, and 75 features were determined according to their rank values. The first 25 (Group 1), second 25 (Group 2), and third 25 (Group 3) attribute groups with the highest rank are listed in Table 6.

The metrics obtained from the classification process using the attributes in Group 1 in Table 6 are listed in Table 7. In total, the accuracy values ranged from 0.64 to 0.90 for the 22 classifiers. The Fine Gaussian SVM method had the lowest accuracy, whereas the Narrow Neural Network had the highest accuracy. The sensitivity value was 0.96, and the recall value was 0.96, with the highest from the Kernel Naive Bayes method. While the Decision Tree performed well in specificity and precision values, the Narrow Neural Network performed better in Fscore and Gmean metrics. Medium and Coarse Gaussian SVM were the best classifiers for the AUC value.

The performance evaluation values, as a result of the analysis in which the best 50 attributes obtained by combining the attributes in Group 1 and Group 2 in Table 6, were used as the inputs listed in Table 8. There are four methods in the table with an accuracy value of 0.9. Although three of these methods were included in the SVM methods, one of them is from the Neural Network family. It can be said that the SVM method’s classifiers gave better performance metrics results than other methods. The specificity, precision, recall, Fscore, and Gmean values were 1.00, 1.00, 0.96, 0.91, and 0.92, respectively. The highest AUC value was seen in the classifier Naive Bayes.

The values in Table 9 were obtained when all of the features in Groups 1, 2, and 3 were included in the analysis. The highest accuracy value was obtained by combining these three groups. The Medium Gaussian SVM method had the highest accuracy value for this feature group, with a value of 0.93. This value was also the highest value among all analyses. The medium Gaussian SVM classifier was the best classifier according to the sensitivity, recall, Fscore, Gmean, AUC, and accuracy values. The highest precision value was seen in Gaussian Naive Bayes, whereas the highest specificity value of 0.94 was seen in Fine Gaussian.

As the number of features used in the analysis increased, the cost and the performance values of many classifiers increased. Although the values of the metrics obtained as a result of Naive Bayes, SVM, and kNN analyses increased close to a linear increase with the increase in the number of features, it was seen that there was an increase in some of the Decision Tree and Neural Network classifiers and a decrease in others. Nevertheless, it can be said that the classifiers included in the study achieved high success in detecting coronary artery disease.

### 3.3. Comparison with Studies in the Literature

The comparative values of the findings in Table 7, Table 8 and Table 9 and the studies on the diagnosis of heart disease from iris images in the literature are listed in Table 10. The table includes the feature extraction methods, classifier names, and evaluation metrics used in existing studies.

Among existing studies, Gunawan et al. [24] obtained 91% accuracy using the SVM classifier with GLCM features. Putra et al. [25] reached an accuracy value of 0.78 by using the Neural Network with the same feature extraction method and also achieved 90% success with the PCA method. Kusuma et al. [27] and Permatasari et al. [26] used the Black and White Ratio and PCA methods for feature extraction, respectively, and performed classification with the Thresholding and SVM methods, respectively. These studies did not include performance metrics other than accuracy. In this study, using wavelet transform-based statistical, GLCM, and GLRLM features and five different classifiers, a higher accuracy value of 93% was obtained with the SVM classifier compared to other studies. In addition, the second highest value was obtained in the NN classifier, with an accuracy value of 92%. In this study, unlike other studies, performance measurements such as sensitivity, specificity, precision, Fscore, Gmean, and AUC were carried out in addition to accuracy. These values indicate that the analysis successfully detected coronary artery disease.

## 4. Conclusions

This study proposes a non-invasive method for detecting coronary artery disease (CAD), as verified in an experiment that used the iris images of 198 volunteers. After the iris pre-processing processes, a total of 136 statistical, GLCM, and GLRLM features were extracted from the four subcomponents obtained by applying wavelet transform to the heart region in the iris. The Relieff feature selection process was used to determine the best 25, 50, and 75 features before classification. The classification phase was carried out using 22 classifiers from five main classifier families. Accuracy, sensitivity, specificity, precision, Fscore, Gmean, and AUC metrics were used to evaluate performance. The SVM Medium Gaussian classifier achieved the highest accuracy value at 93%. According to the results of the other classifiers, it can be said that the CAD classification of the values of accuracy and other metrics yielded successful results. It can be stated that the proposed method for the detection of CAD from the iris is quite successful. The proposed method can be used to support telediagnostic applications for coronary artery disease in telemedicine systems. Thus, information about the patient’s CAD can be obtained by using the patient’s iris images in order to make a preliminary assessment before performing daily clinical practice.

This study provides a reference for detecting CAD from iris images. In future studies, the relationship of various heart diseases, such as heart failure, with iris analysis can be examined. Performance improvement can be made by trying different feature extraction and machine learning methods and by detecting various diseases using convolutional neural networks.

## Figures and Tables

**Table 1:** Age and gender information of volunteers.

Subject	Number of Men	Number of Women	Mean Age	Standard Deviation	Total
Healthy	77	27	55	14.2	104
CAD	79	15	60	9.4	94

**Table 5:** Performance metrics.

Metric	Symbol	Formula
Sensitivity	SNS	$\frac{TP}{TP+FN}$
Specificity	SPC	$\frac{TN}{FP+TN}$
Precision	PRC	$\frac{TP}{TP+FP}$
Accuracy	ACC	$\frac{TP+TN}{TP+FN+FP+TN}$
F_1_ score	F_1_	$2\times \frac{PRC\;x\;SNS}{PRC\;+\;SNS}$
Geometric Mean	GM	$\sqrt{SNS\;x\;SPC}$

**Table 6:** Top 75 features according to the Relieff method.

Group	Feature Numbers
1	103, 105, 31, 28, 32, 5, 70, 127, 126, 135, 35, 134, 98, 102, 24, 69, 33, 30, 34, 37, 125, 29, 107, 66, 116
2	81, 89, 123, 82, 85, 109, 124, 115, 121, 129, 120, 122, 108, 51, 3, 114, 119, 128, 38, 106, 117, 4, 118, 101, 72
3	1, 112, 53, 52, 104, 47, 56, 41, 61, 113, 75, 90, 71, 91, 95, 23, 87, 130, 17, 55, 15, 16, 54, 46, 14

**Table 7:** Results for the best 25 parameters.

Classifiers		Performance Metrics						
		Accuracy	Sensitivity	Specificity	Precision	Fscore	Gmean	AUC
Decision Tree	Fine Tree	0.88	0.84	0.91	0.88	0.86	0.88	0.87
	Medium Tree	0.88	0.84	0.91	0.88	0.86	0.88	0.87
	Coarse Tree	0.83	0.80	0.85	0.80	0.80	0.83	0.86
Naive Bayes	Gaussian	0.85	0.80	0.88	0.83	0.82	0.84	0.95
	Kernel	0.81	0.96	0.71	0.71	0.81	0.82	0.87
SVM	Linear	0.86	0.92	0.82	0.79	0.85	0.87	0.95
	Quadratic	0.88	0.92	0.85	0.82	0.87	0.89	0.92
	Cubic	0.86	0.92	0.82	0.79	0.85	0.87	0.93
	Fine Gaussian	0.64	0.28	0.91	0.70	0.40	0.51	0.75
	Medium Gaussian	0.88	0.92	0.85	0.82	0.87	0.89	0.96
	Coarse Gaussian	0.85	0.84	0.85	0.81	0.82	0.85	0.96
kNN	Fine	0.71	0.72	0.71	0.64	0.68	0.71	0.71
	Medium	0.86	0.88	0.85	0.81	0.85	0.87	0.94
	Coarse	0.85	0.80	0.88	0.83	0.82	0.84	0.95
	Cosine	0.83	0.76	0.88	0.83	0.79	0.82	0.93
	Cubic	0.83	0.80	0.85	0.80	0.80	0.83	0.93
	Weighted	0.85	0.88	0.82	0.79	0.83	0.85	0.94
Neural Network	Narrow	0.90	0.92	0.88	0.85	0.88	0.90	0.91
	Medium	0.83	0.80	0.85	0.80	0.80	0.83	0.9
	Wide	0.86	0.88	0.85	0.81	0.85	0.87	0.9
	Bilayered	0.81	0.84	0.79	0.75	0.79	0.82	0.9
	Trilayered	0.88	0.92	0.85	0.82	0.87	0.89	0.88

**Table 8:** Results for the best 50 parameters.

Classifiers		Performance Metrics						
		Accuracy	Sensitivity	Specificity	Precision	Fscore	Gmean	AUC
Decision Tree	Fine Tree	0.88	0.84	0.91	0.88	0.86	0.88	0.87
	Medium Tree	0.88	0.84	0.91	0.88	0.86	0.88	0.87
	Coarse Tree	0.83	0.80	0.85	0.80	0.80	0.83	0.86
Naive Bayes	Gaussian	0.88	0.88	0.88	0.85	0.86	0.88	0.97
	Kernel	0.83	0.96	0.74	0.73	0.83	0.84	0.87
SVM	Linear	0.88	0.92	0.85	0.82	0.87	0.89	0.96
	Quadratic	0.90	0.92	0.88	0.85	0.88	0.90	0.94
	Cubic	0.90	0.88	0.91	0.88	0.88	0.90	0.96
	Fine Gaussian	0.73	0.36	1.00	1.00	0.53	0.60	0.9
	Medium Gaussian	0.92	0.96	0.88	0.86	0.91	0.92	0.96
	Coarse Gaussian	0.85	0.84	0.85	0.81	0.82	0.85	0.96
kNN	Fine	0.78	0.68	0.85	0.77	0.72	0.76	0.77
	Medium	0.86	0.84	0.88	0.84	0.84	0.86	0.95
	Coarse	0.85	0.76	0.91	0.86	0.81	0.83	0.94
	Cosine	0.86	0.84	0.88	0.84	0.84	0.86	0.96
	Cubic	0.86	0.84	0.88	0.84	0.84	0.86	0.94
	Weighted	0.88	0.88	0.88	0.85	0.86	0.88	0.95
Neural Network	Narrow	0.86	0.92	0.82	0.79	0.85	0.87	0.87
	Medium	0.86	0.88	0.85	0.81	0.85	0.87	0.91
	Wide	0.92	0.96	0.88	0.86	0.91	0.92	0.92
	Bilayered	0.83	0.80	0.85	0.80	0.80	0.83	0.89
	Trilayered	0.88	0.92	0.85	0.82	0.87	0.89	0.89

**Table 9:** Results for the best 75 parameters.

Classifiers		Performance Metrics						
		Accuracy	Sensitivity	Specificity	Precision	Fscore	Gmean	AUC
Decision Tree	Fine Tree	0.83	0.80	0.85	0.80	0.80	0.83	0.84
	Medium Tree	0.83	0.80	0.85	0.80	0.80	0.83	0.84
	Coarse Tree	0.83	0.80	0.85	0.80	0.80	0.83	0.86
Naive Bayes	Gaussian	0.90	0.88	0.91	0.88	0.88	0.90	0.98
	Kernel	0.83	0.92	0.76	0.74	0.82	0.84	0.87
Support Vector Machine	Linear	0.90	0.92	0.88	0.85	0.88	0.90	0.96
	Quadratic	0.88	0.88	0.88	0.85	0.86	0.88	0.95
	Cubic	0.88	0.88	0.88	0.85	0.86	0.88	0.93
	Fine Gaussian	0.69	0.36	0.94	0.82	0.50	0.58	0.91
	Medium Gaussian	0.93	1.00	0.88	0.86	0.93	0.94	0.96
	Coarse Gaussian	0.88	0.88	0.88	0.85	0.86	0.88	0.96
kNN	Fine	0.80	0.72	0.85	0.78	0.75	0.78	0.79
	Medium	0.88	0.88	0.88	0.85	0.86	0.88	0.93
	Coarse	0.80	0.64	0.91	0.84	0.73	0.76	0.94
	Cosine	0.88	0.88	0.88	0.85	0.86	0.88	0.95
	Cubic	0.88	0.88	0.88	0.85	0.86	0.88	0.93
	Weighted	0.90	0.92	0.88	0.85	0.88	0.90	0.94
Neural Network	Narrow	0.85	0.84	0.85	0.81	0.82	0.85	0.89
	Medium	0.85	0.88	0.82	0.79	0.83	0.85	0.89
	Wide	0.85	0.92	0.79	0.77	0.84	0.85	0.91
	Bilayered	0.83	0.88	0.79	0.76	0.81	0.84	0.87
	Trilayered	0.88	0.96	0.82	0.80	0.87	0.89	0.93

**Table 10:** Comparison to existing studies.

References	Feature Extraction	Classifier	Evaluation Metrics						
			Accuracy	Sensitivity	Specificity	Precision	Fscore	Gmean	AUC
Gunawan et al. [24]	GLCM	SVM	0.91	-	-	-	-	-	-
Putra et al. [25]	GLCM	NN	0.78	-	-	-	-	-	-
	PCA	NN	0.90	-	-	-	-	-	-
Kusuma et al. [27]	B&W Ratio	Threshold	0.83	-	-	-	-	-	-
Permatasari et al. [26]	PCA	SVM	0.80	-	-	-	-	-	-
This study	Statistical, GLCM, GLRLM	KNN	0.90	0.92	0.88	0.85	0.88	0.90	0.94
		Naive Bayes	0.90	0.88	0.91	0.88	0.88	0.90	0.98
		SVM	0.93	1.00	0.88	0.86	0.93	0.94	0.96
		DT	0.88	0.84	0.91	0.88	0.86	0.88	0.87
		NN	0.92	0.96	0.88	0.86	0.91	0.92	0.92

## Data Availability

The data that support the findings of this study are available from the corresponding author upon reasonable request.

## References

[1] Virani S.S., Alonso A., Benjamin E.J., Bittencourt M.S., Callaway C.W., Carson A.P., Chamberlain A.M., Chang A.R., Cheng S., Delling F.N. (2020). Heart disease and stroke statistics—2020 update: A report from the American Heart Association. Circulation.

[2] Benjamin E.J., Muntner P., Alonso A., Bittencourt M.S., Callaway C.W., Carson A.P., Chamberlain A.M., Chang A.R., Cheng S., Das S.R. (2019). Heart disease and stroke statistics—2019 update: A report from the American Heart Association. Circulation.

[3] Nowbar A.N., Gitto M., Howard J.P., Francis D.P., Al-Lamee R. (2019). Mortality from ischemic heart disease: Analysis of data from the World Health Organization and coronary artery disease risk factors From NCD Risk Factor Collaboration. Circ. Cardiovasc. Qual. Outcomes.

[4] Malakar A.K., Choudhury D., Halder B., Paul P., Uddin A., Chakraborty S. (2019). A review on coronary artery disease, its risk factors, and therapeutics. J. Cell. Physiol..

[5] Bauersachs R., Zeymer U., Brière J.-B., Marre C., Bowrin K., Huelsebeck M. (2019). Burden of coronary artery disease and peripheral artery disease: A literature review. Cardiovasc. Ther..

[6] Novak R., Hrkac S., Salai G., Bilandzic J., Mitar L., Grgurevic L. (2022). The role of ADAMTS-4 in atherosclerosis and vessel wall abnormalities. J. Vasc. Res..

[7] Ghiasi M.M., Zendehboudi S., Mohsenipour A.A. (2020). Decision tree-based diagnosis of coronary artery disease: CART model. Comput. Methods Programs Biomed..

[8] Alizadehsani R., Zangooei M.H., Hosseini M.J., Habibi J., Khosravi A., Roshanzamir M., Khozeimeh F., Sarrafzadegan N., Nahavandi S. (2016). Coronary artery disease detection using computational intelligence methods. Knowl.-Based Syst..

[9] Fausett L.V. (2006). Fundamentals of Neural Networks: Architectures, Algorithms and Applications.

[10] Jensen B. (2012). Iridology Simplified.

[11] Sivasankar K., Sujaritha M., Pasupathi P., Muthukumar S. FCM based iris image analysis for tissue imbalance stage identification. Proceedings of the 2012 International Conference on Emerging Trends in Science, Engineering and Technology (INCOSET).

[12] Kurnaz Ç., Gül B.K. (2018). Determination of the relationship between sodium ring width on iris and cholesterol level. J. Fac. Eng. Archit. Gazi Univ..

[13] Ma L., Zhang D., Li N., Cai Y., Zuo W., Wang K. (2012). Iris-based medical analysis by geometric deformation features. IEEE J. Biomed. Health Inform..

[14] Samant P., Agarwal R. (2018). Machine learning techniques for medical diagnosis of diabetes using iris images. Comput. Methods Programs Biomed..

[15] Samant P., Agarwal R. (2019). Analysis of computational techniques for diabetes diagnosis using the combination of iris-based features and physiological parameters. Neural Comput. Appl..

[16] Bansal A., Agarwal R., Sharma R. (2015). Determining diabetes using iris recognition system. Int. J. Diabetes Dev. Ctries..

[17] Önal M.N., Güraksin G.E., Duman R. (2022). Convolutional neural network-based diabetes diagnostic system via iridology technique. Multimed. Tools Appl..

[18] Rehman M.U., Najam S., Khalid S., Shafique A., Alqahtani F., Baothman F., Shah S.Y., Abbasi Q.H., Imran M.A., Ahmad J. (2021). Infrared sensing based non-invasive initial diagnosis of chronic liver disease using ensemble learning. IEEE Sens. J..

[19] Muzamil S., Hussain T., Haider A., Waraich U., Ashiq U., Ayguadé E. (2020). An intelligent iris based chronic kidney identification system. Symmetry.

[20] Hernández F., Vega R., Tapia F., Morocho D., Fuertes W. Early detection of Alzheimer’s using digital image processing through iridology, an alternative method. Proceedings of the 2018 13th Iberian Conference on Information Systems and Technologies (CISTI).

[21] Ozbilgin F., Kurnaz C. (2022). An alternative approach for determining the cholesterol level: Iris analysis. Int. J. Imaging Syst. Technol..

[22] Özbilgin F. (2019). Determination of Iris Symptoms of Systemic Diseases by Iris Analysis Method. Master’s Thesis.

[23] Ramlee R., Ranjit S. Using iris recognition algorithm, detecting cholesterol presence. Proceedings of the 2009 International Conference on Information Management and Engineering.

[24] Gunawan V.A., Putra L.S.A., Imansyah F., Kusumawardhani E. (2022). Identification of Coronary Heart Disease through Iris using Gray Level Co-occurrence Matrix and Support Vector Machine Classification. Int. J. Adv. Comput. Sci. Appl..

[25] Putra L.S.A., Isnanto R.R., Triwiyatno A., Gunawan V.A. Identification of Heart Disease with Iridology Using Backpropagation Neural Network. Proceedings of the 2018 2nd Borneo International Conference on Applied Mathematics and Engineering (BICAME).

[26] Permatasari L.I., Novianty A., Purboyo T.W. Heart disorder detection based on computerized iridology using support vector machine. Proceedings of the 2016 International Conference on Control, Electronics, Renewable Energy and Communications (ICCEREC).

[27] Kusuma F.D., Kusumaningtyas E.M., Barakbah A.R., Hermawan A.A. Heart abnormalities detection through iris based on mobile. Proceedings of the 2018 International Electronics Symposium on Knowledge Creation and Intelligent Computing (IES-KCIC).

[28] Daugman J. (2009). How iris recognition works. The Essential Guide to Image Processing.

[29] Reza A.M. (2004). Realization of the contrast limited adaptive histogram equalization (CLAHE) for real-time image enhancement. J. VLSI Signal Process. Syst. Signal Image Video Technol..

[30] Diwakar M., Tripathi A., Joshi K., Sharma A., Singh P., Memoria M. (2021). A comparative review: Medical image fusion using SWT and DWT. Mater. Today Proc..

[31] Kumar S., Singh B.K. (2021). DWT based color image watermarking using maximum entropy. Multimed. Tools Appl..

[32] Haralick R.M., Shanmugam K., Dinstein I.H. (1973). Textural features for image classification. IEEE Trans. Syst. Man Cybern..

[33] Soh L.-K., Tsatsoulis C. (1999). Texture analysis of SAR sea ice imagery using gray level co-occurrence matrices. IEEE Trans. Geosci. Remote Sens..

[34] Clausi D.A. (2002). An analysis of co-occurrence texture statistics as a function of grey level quantization. Can. J. Remote Sens..

[35] Kira K., Rendell L.A. (1992). A practical approach to feature selection. Machine Learning Proceedings 1992.

[36] Kononenko I. Estimating attributes: Analysis and extensions of RELIEF. Proceedings of the European Conference on Machine Learning.

[37] Luque A., Carrasco A., Martín A., de Las Heras A. (2019). The impact of class imbalance in classification performance metrics based on the binary confusion matrix. Pattern Recognit..

[38] Chicco D., Tötsch N., Jurman G. (2021). The Matthews correlation coefficient (MCC) is more reliable than balanced accuracy, bookmaker informedness, and markedness in two-class confusion matrix evaluation. BioData Min..

[39] Room C. (2019). Confusion Matrix. Mach. Learn.

[40] Hasan M.K., Alam M.A., Das D., Hossain E., Hasan M. (2020). Diabetes prediction using ensembling of different machine learning classifiers. IEEE Access.

